# WHO, RECIST, and immune-related response criteria: is it time to revisit pembrolizumab results?

**DOI:** 10.3332/ecancer.2015.604

**Published:** 2015-12-03

**Authors:** Felipe Ades, Nise Yamaguchi

**Affiliations:** Centro de Oncologia e Hematologia, Hospital Israelita Albert Einstein, Avenida Albert Einsten, 627/701, 3º SS—Morumbi, São Paulo—SP, CEP05652–900, Brazil

**Keywords:** pembrolizumab, RECIST, irRC, immune-related response criteria, WHO response criteria, melanoma

## Abstract

In recent years, with the rise of immunotherapeutic agents for cancer treatment, we have observed a paradigm shift in oncology drug development. One common problem accompanying such paradigm shifts is how to build research strategies to fit the mechanism of action of the newer compounds. Developing immunotherapy in oncology requires us to address the unique characteristics of immunotherapeutic agents and to provide adequate tools for their evaluation, including the adjustment of clinical trial endpoints. Immunotherapy creates patterns of response different from those of chemotherapy, and thus they are not captured by the traditional World Health Organisation (WHO) tumour response criteria or the RECIST. Revisiting the results of pembrolizumab in patients with melanoma can help to evaluate the efficacy of the immune-related response criteria (irRC) as the gold standard for evaluating the clinical response of immunologic agents in oncology.

In 1962, a physicist introduced a concept that changed the way we perceive science. In his book ‘The Structure of Scientific Revolutions’, Thomas Kuhn postulated that science does not progress in a continuous linear way but instead undergoes periodic revolutions, which he named ‘paradigm shifts’ [1]. In recent years, with the rise of immunotherapeutic agents for cancer treatment, we have observed a paradigm shift in oncology drug development.

One common problem accompanying such paradigm shifts is how to build research strategies to fit the mechanism of action of the newer compounds. Standard drug development models and evaluation scales were created for–and well suited to–cytotoxic drugs. These drugs act by killing cancerous cells, and if effective can cause fast reduction in tumour size. The problem is that this is not the mechanism of action of many innovative drugs currently in development, including immunologic agents [2]. As stated by Hoos and colleagues, developing immunotherapy in oncology requires us to address the unique characteristics of immunotherapeutic agents and to provide adequate tools for their evaluation, including the adjustment of clinical trial endpoints [3].

The *in vivo* effects of immunotherapies can be divided conceptually into three phases. After a drug administration, T-cell proliferation and immune activation is observed. Thereupon over a period of weeks or months, the clinical effect of a drug can be measured by reduction in tumour size and improvement in patient’s performance. Finally, this can give rise to delayed effect on a patient’s survival, i.e. several months following the drug is administered. Immunotherapy creates patterns of response different from those of chemotherapy [3], and thus they are not captured by the traditional World Health Organisation (WHO) tumour response criteria or the RECIST (Response evaluation criteria in solid tumours) [4].

To address the need for new tools to evaluate the clinical activity of immunotherapeutic agents, a consortium of approximately 200 oncologists, immunotherapists, and regulatory experts joined forces in 2004 and 2005. This led to the development of the immune-related response criteria (irRC) [5] aiming at capturing patterns of tumour response beyond those seen with cytotoxic agents. These assess tumour burden as a continuous variable, allowing the evaluation of percentage changes in several target lesions overtime. Hence by doing so, they capture the growth kinetics of the total measurable tumour burden [5]. One of the most important differences is the concept of tumour burden and not only that of target lesions. It incorporates changes in all lesions to define the response pattern. The appearance of new lesions are evaluated in the context of all disease and not considered progressive disease *per se*. The irCR has also higher thresholds to determine progression or response, 25% increase and 50% decrease respectively; in comparison to RECIST that uses 20% increase and 30% decrease respectively. Table 1 compares the RECIST, the WHO, and the irRC criteria.

Pembrolizumab (formerly lambrolizumab) is monoclonal IgG4 antibody targeting the PD-1 receptor. Linking the T-cell PD-1 receptor with PD-L1, expressed by many tumour cells leads to a negative regulation of immune response. Pembrolizumab acts by avoiding this interaction, thus increasing T-cell mediated antitumour activity.

In a recent study performed by Hamid and colleagues, two doses of pembrolizumab were administered to a total of 276 patients with melanoma in two cohorts, ipilimumab-pretreated or ipilimumab-naïve. The study found that the two doses of pembrolizumab (2 mg/kg or 10 mg/kg) had similar efficacy and safety profiles in both cohorts [6].

One interesting finding of this study was the comparison of the efficacy results using the RECIST and irRC criteria. Using RECIST, the overall response rates at 2 mg/kg and 10 mg/kg were 33% and 40% respectively among the ipilimumab-naïve patients, and 26% for both doses in the ipilimumab-pretreated group. Analysing the results using the irRC criteria, these figures were 39% and 40% in the naïve group and 27% and 32% in the pretreated group respectively. Similar results occur in the analysis of progression-free survival (PFS): no difference was seen between the two dose groups. However, the analysis by irRC detected a higher number of responders: the 24-week PFS rate in the naïve cohort was 48% and 50% for the two dose levels respectively according to RECIST, and 50% and 60% for irRC. In the pretreated cohort, these figures were 37% and 44% for the two dose levels respectively according to RECIST, and 57% for both dose levels by irRC [6].

Bearing the difference of these results in mind, we would like to draw attention to a previous publication by Hamid and colleagues in the *New England Journal of Medicine*. In this study the investigators administered 10 mg/kg of pembrolizumab every two or three weeks or 2 mg/kg every three weeks to a similar group of patients. The results were assessed every 12 weeks according to the RECIST criteria [7].

The overall response rate was 38% (95% confidence interval [CI], 25–44), with the highest confirmed response rate observed in the 10 mg/kg cohort every two weeks (52%; 95% CI, 38–66). The response rate was similar across the ipilimumab-naïve or pretreated cohorts (38% [95% CI, 23–55] and 37% [95% CI, 26–49] respectively). Long lasting responses were seen in the majority of patients (median follow-up 11 months among patients who had shown a response), and a total of 81% of the patients who responded were still on treatment at the time of the publication [7].

This study provides another opportunity to evaluate the efficacy of irRC as response criteria. Revisiting this analysis in the framework of irRC can provide new insights into the real effect of pembrolizumab in patients with melanoma and can help to consolidate these criteria as the gold standard for evaluating the clinical response of immunologic agents in oncology. We congratulate the authors for their two important studies, and we would like to invite them to present an update of the results of their work according to the irRC criteria.

## Figures and Tables

**Table 1.** Comparison between the RECIST 1.1, the WHO and the irRC criteria (adapted from Wolchok 2009).

Comparison between RECIST, WHO, and irRC criteria			
	RECIST	WHO	irRC
**New, measurable lesions (i.e. ≥5 × 5 mm)**	Always represent PD	Always represent PD	Incorporated into tumour burden
**New, non-measurable lesions (i.e. <5 × 5 mm)**	Always represent PD	Always represent PD	Do not define progression (but preclude irRC)
**Non-index lesions**	Changes contribute to defining BOR of CR, PR, SD, and PD	Changes contribute to defining BOR of CR, PR, SD, and PD	Contribute to defining irRC (complete disappearance required)
**Complete response (CR)**	Disappearance of all lesions in one observation in randomised studies. Confirmation is needed for non-randomised studies, according to study protocol	Disappearance of all lesions in two consecutive observations not less than four weeks apart	Disappearance of all lesions in two consecutive observations not less than four weeks apart
**Partial response (PR)**	At least a 30% decrease in the sum of diameters of target lesions, taking as reference the baseline sum diameters, in the absence of new lesions or unequivocal progression of non-index lesions	A ≥50% decrease in SPD of all index lesions compared with baseline in two observations at least four weeks apart, in the absence of new lesions or unequivocal progression of non-index lesions	A ≥50% decrease in tumour burden compared with baseline in two observations at least four weeks apart
**Stable disease (SD)**	Neither sufficient shrinkage to qualify for PR nor sufficient increase to qualify for PD, taking as reference the smallest sum diameters, in absence of new lesions or unequivocal progression of non-index lesions	A 50% decrease in SPD compared with baseline cannot be established nor 25% increase compared with nadir, in absence of new lesions or unequivocal progression of non-index lesions	A 50% decrease in tumour burden compared with baseline cannot be established nor 25% increase compared with nadir
**Progressive disease (PD)**	At least a 20% increase in the sum of diameters of target lesions, taking as reference the smallest sum on study. The sum must also demonstrate an absolute increase of at least 5 mm. The appearance of one or more new lesions is also considered progression	At least 25% increase in SPD compared with nadir and/or unequivocal progression of non-index lesions and/or appearance of new lesions (at any single time point)	At least 25% increase in tumour burden compared with nadir (at any single time point) in two consecutive observations at least four weeks apart

RECIST : Response evaluation criteria in solid tumours; WHO: World Health Organisation; irRC: immune-related response criteria; PD: progressive disease; BOR: best overall response; CR: complete response; PR: partial response; SD: stable disease; PD: progressive disease; SPD: sum of the product of the greatest diameters.
